# Supplementary material for: Anti-Influenza with Green Tea Catechins: A Systematic Review and Meta-Analysis
Source: Molecules. 2021 Jun 30;26(13):4014. doi: 10.3390/molecules26134014 (PMC8272076; doi:10.3390/molecules26134014)
Supplement: Supplementary file 1 [file molecules-26-04014-s001.zip › molecules-1278243-supplementary/Supplemental/Table S1.pdf]

Table S1. Event of type of influenza virus infection and EGCG consumption concentrations

Authors (year)	Type of influenza virus				EGCG (mg/day)	
	A	B	A and B	A or B	≤ 338	>338
Yamada et al (2006) [37]	0	6	0	0	76	0
Rowe et al (2007) [32]	0	0	0	53	0	53
Yamada et al (2007) [33]	6	0	0	0	395	0
Matsumoto et al (2011) [34]	6	11	0	0	97	0
Park et al (2011) [38]	185	18	1	0	1954	0
Toyoizumi et al (2013) [35]	0	0	0	23	155	0
Ide et al (2014) [36]	42	2	0	0	384	0
Delabre et al (2015) [39]	44	37	8	0	193	0

Abbreviations: EGCG, Epigallocatechin gallate.
