# Supplementary material for: Anti-Influenza with Green Tea Catechins: A Systematic Review and Meta-Analysis
Source: Molecules. 2021 Jun 30;26(13):4014. doi: 10.3390/molecules26134014 (PMC8272076; doi:10.3390/molecules26134014)
Supplement: Supplementary file 1 [file molecules-26-04014-s001.zip › molecules-1278243-supplementary/Supplemental/Table S2 ROBINS-I.pdf]

Table S2 The Risk Of Bias In Non-randomized Studies of Interventions (ROBINS-I) tool

	Confounding domain	Selection of participants into the study	Classification of interventions	Deviation from intended interventions	Missing data	Measurement of outcomes	Election of reported result	Overall quality
Yamada 2006	M	L	L	N	N	L	L	M
Park 2011	M	L	L	S	N	S	L	S
Delabre 2014	M	L	L	L	C	L	L	C

Critical	Serious	Moderate	Low	No information
