# Supplementary figures and images for: Anti-Influenza with Green Tea Catechins: A Systematic Review and Meta-Analysis
Source: Molecules. 2021 Jun 30;26(13):4014. doi: 10.3390/molecules26134014 (PMC8272076; doi:10.3390/molecules26134014)

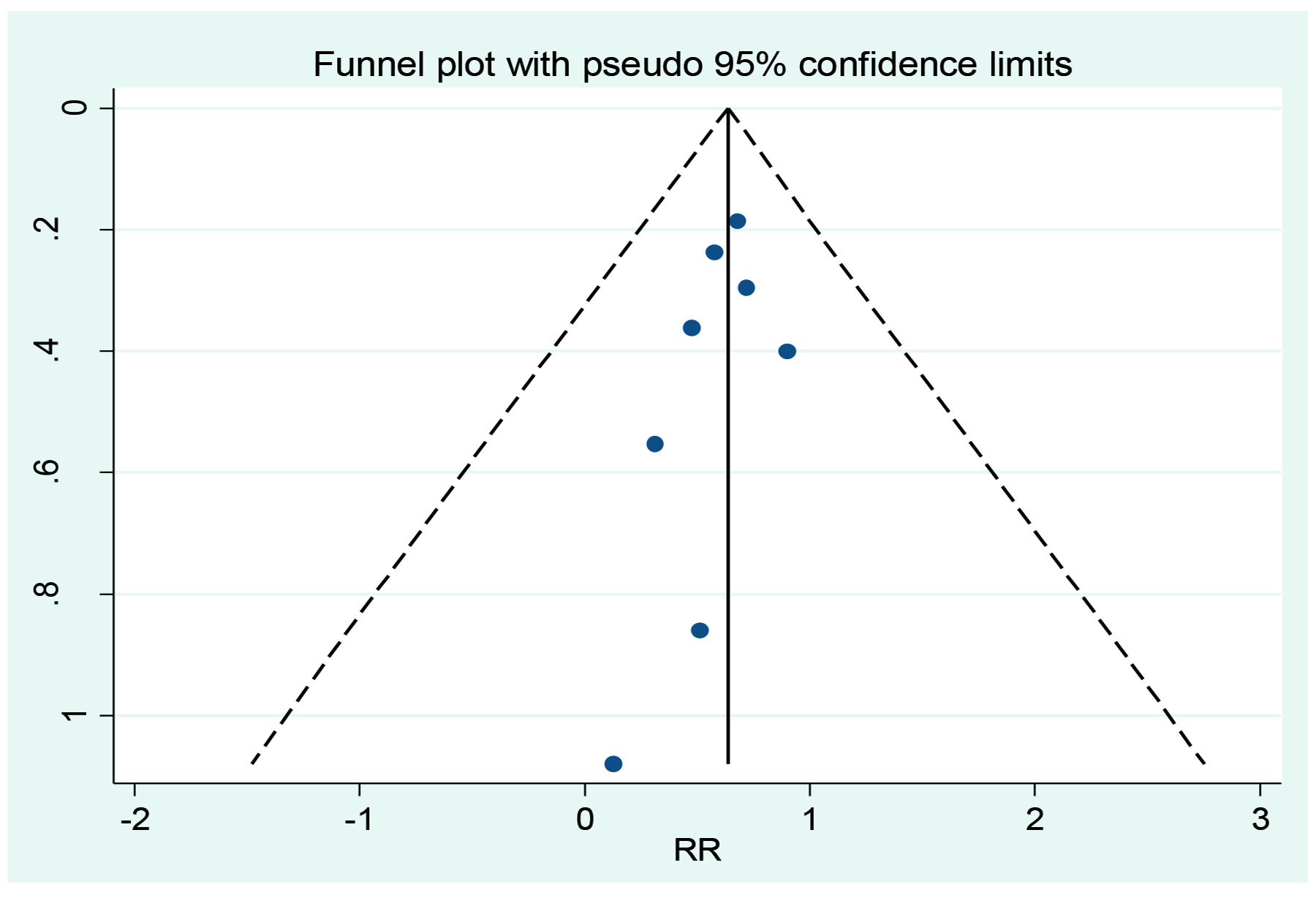

Supplement: Supplementary file 1 [file molecules-26-04014-s001.zip › molecules-1278243-supplementary/Supplemental/Fig S1.tif]
